# Cyclin G2 Inhibits Oral Squamous Cell Carcinoma Growth and Metastasis by Binding to IGFBP3 and Regulating the FAK-SRC-STAT Signaling Pathway

**DOI:** 10.3389/fonc.2020.560572

**Published:** 2020-11-06

**Authors:** Danning Wang, Jinlan Gao, Chenyang Zhao, Sen Li, Di Zhang, Xiaoyu Hou, Xinbin Zhuang, Qi Liu, Yang Luo

**Affiliations:** The Research Center for Medical Genomics, Key Laboratory of Cell Biology, Key Laboratory of Medical Cell Biology, Ministry of Education, School of Life Sciences, China Medical University, Shenyang, China

**Keywords:** cell cycle protein cyclin G2, oral squamous cell carcinoma, tumor suppressor, insulin-like growth factor binding protein 3, integrin, focal adhesion kinase, c-Src

## Abstract

The cell cycle protein cyclin G2 is considered a tumor suppressor. However, its regulatory effects and potential mechanisms in oral cancers are not well understood. This study aimed to investigate the effect of cyclin G2 on oral squamous cell carcinoma (OSCC). The data from 80 patients with OSCC were utilized to predict the abnormal expression of cyclin G2. The proliferation and metastasis were determined by a cell counting Kit-8 assay, flow cytometry, a wound-healing assay, and a cell invasion assay. The expression of key proteins and genes associated with the cyclin G2 signaling pathways was determined by western blotting and real-time PCR, respectively. The orthotopic nude mice model was established by a mouth injection of SCC9 cells overexpressing cyclin G2. We showed that the low level of cyclin G2 in OSCC, which is negatively correlated with clinical staging, was a negative prognostic factor for the disease. We also found that cyclin G2 inhibited the proliferation, metastasis, and blocked the cell cycle at G1/S of OSCC cells, suggesting that cyclin G2 has an inhibitory effect in OSCC. Mechanistically, cyclin G2 inhibited the growth and metastasis of OSCC by binding to insulin-like growth factor binding protein 3 (IGFBP3) and regulating the focal adhesion kinase (FAK) -SRC-STAT signal transduction pathway. Cyclin G2 competed with integrin to bind to IGFBP3; the binding between integrin and IGFBP3 was reduced after cyclin G2 overexpression, thereby inhibiting the phosphorylation of FAK and SRC. These results showed that cyclin G2 inhibited the progression of OSCC by interacting with IGFBP3 and that it may be a new target for OSCC treatment.

## Introduction

Oral squamous cell carcinoma (OSCC) is the sixth most common malignant tumor in the world and the most common malignant oral cancer tumor, accounting for more than 90% of oral cancer cases. More than 300,000 new cases are diagnosed each year, and the morbidity and mortality among young people have continually risen in recent year (1, 2). Despite recent advances in radiotherapy, chemotherapy, and traditional surgery, the 5-year survival rate for OSCC is still only about 50% (3). Although the cure rate of early OSCC exceeds 80%, more than 70% of patients with advanced OSCC cannot be cured (4, 5).It is generally believed that OSCC has a high potential for local invasion and lymph node metastasis, and clinical staging plays an important role in the survival prediction of OSCC patients (6).Nonetheless, the precise mechanisms leading to OSCC are not fully understood.

Cyclin G2 is an unconventional cell cycle protein encoded by the *CCNG2* gene that plays a negative role in the cell cycle process (7).Thus, it also plays an important role in the biological functions of cell growth inhibition and tumor suppression (8). Cyclin G2 is a potential cancer suppressor gene whose expression may be associated with the pathological process underlying tumor development (9).The expression levels of cyclin G2 are reduced in thyroid, breast, kidney, stomach, esophageal, pancreatic, and other tumors according to early reports (7, 8, 10–13). Anticancer drugs have also been shown to increase the level of cyclin G2 to induce arrest, knockdown of cyclin G2 reduces the potency of cotylenin A and rapamycin (14). The precise mechanism of action of cyclin G2 has not been elucidated there are reports that speculate on the mechanisms by which cyclin G2 is expressed (15). However, the exact role that cyclin G2 plays in OSCC and other cancers is still unknown.

Thus, the goal of our research was to explore the relationship between cyclin G2 and the growth and metastasis in OSCC and the underlying molecular mechanisms. The patient data showed a significant decrease in cyclin G2 expression through advanced cancer stages. On conducting a mass spectrometric analysis, we were able to identify that IGFBP3 was a possible cyclin G2 interacting protein. It is known that IGFBP3 promotes OSCC cell migration and lymph node metastasis through integrin β1 in an insulin-independent manner (16). It has also been reported that inside-out signaling through the integrin-FAK axis may regulate cancer cell adhesion, proliferation, and metastasis (17). Furthermore, we found that the combination between integrin and IGFBP3, the phosphorylation of FAK and SRC and the FAK-SRC-STAT axis were negatively regulated after cyclin G2 overexpression. Taken together, these results indicated that cyclin G2 functioned as a tumor suppressor in OSCC through its interaction with IGFBP3 and the subsequent blockage of the connection between integrin and IGFBP3, the phosphorylation of FAK and the FAK-SRC-STAT signaling pathways. When the phosphorylation of STAT3 was reduced, its translocation to the nucleus was reduced, which decreased the transcriptional regulation of *Bcl-2*, *c-Myc*, and *MMP9*, thereby regulating cell growth and metastasis. STAT3 is constitutively active in a variety of cancers. This activation deregulates the signaling that controls cell proliferation, including signals involving Bcl-2 and c-Myc, and metastasis and angiogenesis, which involves MMP-9. This deregulation promotes tumor progression (17). This study demonstrated the inhibitory function of cyclin G2 in OSCC growth and metastasis and explored the underlying mechanisms. Thus, Cyclin G2 maybe a key treatment target of OSCC.

## Methods

### Patients and Tissue

The tissue samples were collected from 80 patients diagnosed with OSCC who received treatment at the Oral Hospital of China Medical University between January 2018 and March 2019. The patients underwent extensive OSCC resection during the surgery, and OSCC tissue and paired normal tissue were obtained for postoperative analysis. In addition, clinical data were collected, including the patients’ age and sex, tumor size, metastasis status, and clinical stage.

### Hematoxylin and Eosin Staining and Immunohistochemistry

The OSCC tissues from each group were paraffin embedded and were cut on a microtome into 4 µm sections. The tissues were stained using a hematoxylin-eosin staining kit (Wanlei, Shenyang, China) and were observed under a microscope (Olympus, CKX41 Tokyo, Japan). In addition, some of the tissue sections were dewaxed in xylene, rehydrated with alcohol, treated for antigen-retrieval, incubated in hydrogen peroxide, blocked with goat serum, and then were incubated with one of the following primary antibodies: anti-cyclin G2 antibody (HPA034684, Sigma -Aldrich, Santa Clara, CA, USA); phospho-FAK (ab81298, Tyr397, Abcam, Cambridge, MA, USA); phospho-SRC (AF3162, Tyr419, Affinity Biosciences, OH, USA) or phospho-STAT3 (AF3293, Tyr705, Affinity) overnight at 4°C. The membranes were incubated with an HRP-linked secondary antibody [Goat anti-Rabbit IgG (H+L), 31460, Thermo Fisher, Waltham, MA, USA], DAB stained, hematoxylin counter stained and were imaged under the microscope. A staining index (0–12) was determined by multiplying the score of the staining intensity by the score for the positive cells. The intensity was scored as follows: 0, negative; 1, weak; 2, moderate and 3, strong. The positive cell frequency was scored as follows: 0, less than 5%; 1, 5–25%; 2, 26–50%; 3, 51–75% and 4, greater than 75%.

### Cell Culture

The human OSCC cell line SCC-9 was purchased from Cobioer (Nanjing, China), and the cells were cultured in DMEM/F12 medium (including 10% fetal bovine serum and 0.4 µg/ml hydrocortisone). The cells were maintained in an incubator at 37°C and with 5% CO_2_.

### Cell Infection

To generate cell lines that stably overexpressed cyclin G2, FLAG-tagged cyclin G2 (FLAG-*CCNG2*) was cloned into a lentivirus-GFP lentiviral vector and waspackaged by GeneChem Co., Ltd (Shanghai, China) used as previously described (18). A GFP-lentiviral vector was used as a negative control. The SCC-9 cells were plated at a density of 1 × 10^5^ cells per well in 24-well plates. When the cell confluence reached 70%, the cells were infected with the cyclin G2 overexpressing and negative control lentiviral vectors. The transduced cells were selected with puromycin.

### Cell Proliferation Assay

The cells were plated in 96-well plates at a density of 5 × 10^3^ cells per well for 0, 24, 48, and 72 h. CCK8 (Beyotime Biotechnology, Shanghai, China) was added to each well, and the plates were incubated for 2 h at 37°C. The absorbance was measured at 490 nm using an ultraviolet spectrophotometer (Thermo Fisher Scientific, Waltham, MA, USA).

### Cell Cycle Detection

A total of 5 × 10^5^ cells was collected and fixed in 70% ethanol overnight and were then incubated with a propidium iodide stain (BD Biosciences, Franklin Lakes, NJ, USA) at 4°C. The percentage of cells in the different cell cycles was then determined on flow cytometer NOVOcyte ACEA biosciences Inc. USA.

### Wound-Healing Assay

The cells were plated at a density of 5 × 10^5^ per well in a 6-well plate. Then, a 2-mm wide plastic pipette tip was used to scrap the cells to perform wound healing assays. The cells were cultured in a serum-free medium and observed and imaged under a microscope at 0 and 24 h. The wound-healing rate was calculated as (the 0 h cell wound width—the experimental point cell wound width)/0 h cell wound width × 100%.

### Cell Invasion Assay

The chambers (8µm pore size, corning, NY, USA) were pre-coated with Matrigel (BD Biosciences, NJ, USA 356234). The cell concentration was adjusted to 5 × 10^4^/ml in serum-free medium, and 200 μl of cell suspension was seeded to the upper chamber; complete culture medium was added to the lower layer. After 24 h in culture, the upper matrix material was removed using a cotton swab, and the cells were fixed and stained with crystal violet. Then, 5 randomly chosen fields were counted to determine the cell migration indices, and these were imaged under a microscope.

### Production of MMP9

The cells were plated at a density of 5 × 10^4^ per well in 24-well plates. The supernatant was collected after the 24 and 48 h culture, and MMP9 levels were determined using an MMP9 ELISA kit (Boster Biosciences, Wuhan, China AB246539). The methods were as described in the manufacturer’s protocol. The supernatants were collected, and the MMP9 concentrations were measured at 450 nm. The values were calculated using a standard curve.

### Mass Spectrometry

The mass spectrometry was performed according to the methods reported in our previous study (19). The cells expressed FLAG or FLAG-tagged cyclin G2 was lysed using RIPA lysis buffer. The immunoprecipitation assay was performed to separate the FLAG-tagged protein from the lysate using the anti-FLAG affinity gel. After the anti-FLAG affinity gel was washed in PBS and resuspended in 2×loading buffer, the proteins were subjected to polyacrylamide gel and stained with Coomassie Brilliant Blue (R250) (6104-59-2). The aim bands were harvested and digested to conduct mass spectrometry.

### Co-Immunoprecipitation and Western Blot

The cell lysates were extracted with RIPA lysis buffer that contained a protease inhibitor cocktail and phosphatase inhibitor cocktail (Roche, Basel, Switzerland). Equivalent concentrations of protein were separated using gel electrophoresis and were then transferred to a PVDF membrane using wet transfer. The membranes were incubated with one of the following primary antibodies: anti-cyclin G2 (1:500, HPA034684, Sigma); Flag (1:500, ab49763, Abcam); Integrin β1 (1:1,000, ab52971, Abcam); FAK (1:1,000, bs-1340R, Bioss, Beijing, China); phospho-FAK (1:1,000, ab81298, Tyr397, Abcam); SRC (1:500, WL01570, Wanlei); phospho-SRC (1:1,000, AF3162, Tyr419, Affinity); STAT3 (1:300, WL01836, Wanlei); phospho-STAT3 (1:1,000, AF3294, Affinity); Bcl-2 (1:1,000, ab32124, Abcam); c-Myc (1:500, 9E10, Santa Cruz Biotechnology, CA, USA); MMP9 (1:500, 10375-2-AP, Proteintech, ID, USA); β-actin (1:1,000, ab6276, Abcam) and GAPDH (1:1,000, 5174, Cell Signaling Technology, Danvers, MA, USA). For at least 6 h and then were washed with PBST to remove excess antibody. The membranes were then incubated for 2 h with the appropriate secondary antibody. The membrane was washed with PBST to remove excess secondary antibody. The protein levels were determined using an ECL Plus kit For the Co-IP experiment 80–90% confluent cells were lysed and protein was extracted. 40 μl of protein A/G magnetic beads were taken in 400 μl PBST and separated them using a magnetic stand. The supernatant was discarded and the beads were washed 3 times. 400 μl of IGFBP3 antibody was added which was diluted to 1:2,000, they were mixed for 2 h and washed with PBST. Equal amounts of protein lysate from the cells and magnetic beads were incubated overnight. They were then washed with PBST and SDS PAGE Followed by Western Blot was performed.

### RT-qPCR

After cell lysis, TRIzol (Invitrogen) was added to extract the RNA, and high-fidelity enzymes were used to convert it into cDNA. The following primers were designed: CCNG2 F: 5’ CGGAGAATGATAACACTTTG, *CCNG2* R: 5’ GTTTCACCTTCATAAGAGCC; *IGFBP3* F: 5’ AAATGCTAGTGAGTCGGAGGAA, *IGFBP3* R: 5’ GATGATTATCTTTGAATGGAGGG; *c-Myc* F: 5’ CACCCTTCTCCCTTCGG, *c-Myc* R: 5’ CAGTCCTGGATGATGATGTTT; *Bcl2* F: 5’ TGTGGCCTTCTTTGAGTTCG, *Bcl-2* R: 5’ CATCCCAGCCTCCGTTATCC; *MMP9* F: 5’ TTTGACAGCGACAAGAAGTG, *MMP9* R: 5’ CAGGGCCGAGGACCATAGAGG and *GAPDH* F: 5’ TGTTGCCATCAATGACCCCTT, *GAPDH* R: 5’ TCCACGACGTACTCAGCG. The primers were synthesized by Sangon Biotech (Shanghai, China). The cDnaalong with the primers was amplified using SYBEr green master mix and Thermo Scientific ABgene^®^ qPCR system.

### Fluorescence Microscopy

Cells grown on coverslips were incubated overnight at 4°C with primary antibody against anti-cyclin G2 antibody (HPA034684, Sigma -Aldrich, Santa Clara, CA, USA), followed by incubation for 45 min at 37°C with Goat anti-Rabbit IgG (H+L) (31460, Thermo Fisher, Waltham, MA, USA). Slides were counterstained with DAPI to visualize the cell nuclei, photographed using an Olympus LEXT OLS4500 confocal laser scanning microscope.

### Animal Experiments

BALB/C nude mice (5- to 6-week-old females, 18–20 g) were purchased from Beijing Vital River Laboratory Animal Technology Co., Ltd. The mice were randomly divided into two groups (n = 6/group). After abdominal cavity anesthesia with 1% sodium pentobarbital at a concentration of 500 mg/kg, a total of 1 × 10^7^ SCC9 cells overexpressing cyclin G2 or control GFP (NC) cells in 50 µl of PBS was injected into the tongue of the nude mice (20). Mice were housed at room with 40–60% humidity, and with a light cycle of 12 h light and 12 h dark under pathogen-free conditions. Animal health and behavior were monitored every day. All of the mice were maintained using a homemade semi-flow feeding system, which refers to the recipe of rodent diet (21) and contained 35% ewe’s milk, 35% corn starch, 10% gelatin, 10% eggs, 5% corn oil, and a 5% sugar and vitamin mix. One mouse lost weight and starved to death on the 20th day, so 12 mice (6 pairs) were included in the end. All of the twelve mice were euthanized *via* anesthesia overdose in 42 days after injection (16), and the tumors were isolated. We then measured the weight and volume using the formula V = length*width*height, and also extracted the swollen lymph nodes, followed by fixing and embedding.

### Statistical Analysis

All the experiments were repeated at least three times, and the results are expressed as the mean ± standard deviation. SPSS 19.0 software was used for the t-test, chi-square test and ANOVA. P <0.05 was considered to indicate statistical significance.

## Results

### Cyclin G2 Expression Is Decreased in Human OSCC Tissue

**Figure 1:**
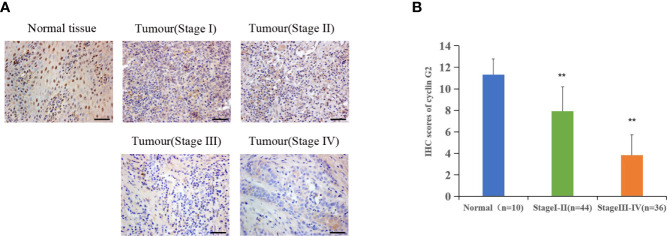
Cyclin G2 expression negatively correlates with clinical stage in OSCC. **(A)** The expression of cyclin G2 in OSCC at the different clinical stages by an immunohistochemical analysis. Scale bar = 100 µm. **(B)** Immunohistochemical scores of cyclin G2 at the different clinical stages. **p < 0.01 vs. Normal group.

Immunohistochemistry was performed on 80 OSCC tissue samples and 10 adjacent normal tissue samples to assess cyclin G2 expression, and the results were analyzed in combination with the clinical data. Age, sex, and tumor size were not correlated with the expression of cyclin G2, whereas the clinical stage was associated with cyclin G2 expression (P < 0.05). The expression of cyclin G2 was significantly reduced in the stage III and IV samples and was negatively correlated with the clinical stage (**Table 1**, **Figures 1A, B**).

**Table 1:** Relationship between positive immunohistochemical expression of cyclin G2 and the clinical characteristics of oral squamous cancer.

Clinicopathologic features	No. of cases	IHC scores of cyclin G2	*p* value
Age			
>60	49	6.28±2.95	0.781
≤60	31	6.05±2.93	
Gender			
Male	46	5.86±3.02	0.182
Female	34	6.88±2.58	
Tumor size (cm^3^)			
>4	11	6.54±1.72	0.087
≤4	69	6.27±2.81	
Metastasis status			
Yes	25	3.48±1.92	0.007
No	55	6.94±2.12	
TNM stage			
Normal tissue	10	11.3±1.49	*p*<0.001
Tumor (Stage I–II)	44	7.94±2.24	
Tumor (Stage III–IV)	36	3.85±1.87	

### Cyclin G2 Inhibits the Viability and Metastasis of OSCC Cells and Blocks the Cell Cycle at G1/S

**Figure 2:**
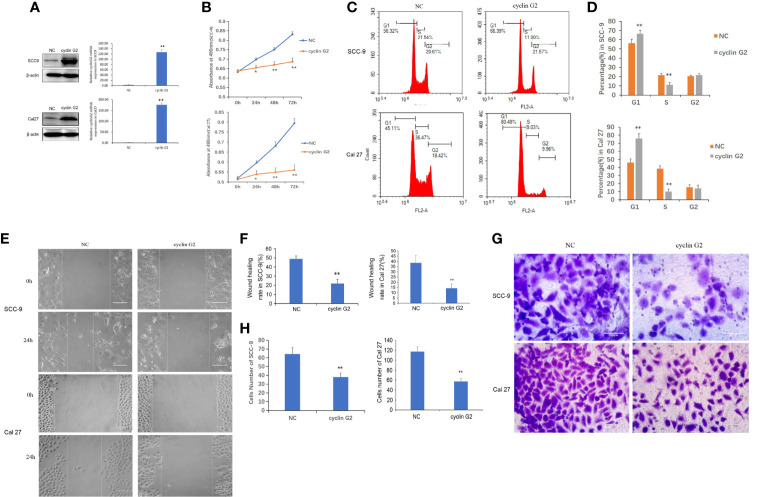
Cyclin G2 inhibits the proliferation and metastasis of OSCC cells and blocks the cell cycle at G1/S. **(A)** The expression of cyclin G2 protein and mRNA levels in NC groups and cyclin G2 overexpressing groups in SCC-9 and Cal 27 cell lines. **(B)** Absorbance of the SCC-9 and Cal 27 cells in the CCK8 cell proliferation experiment. **(C, D)** Flow cytometry detection of the cell cycle in the SCC-9 and Cal 27 cells. **(E, F)** Representative photos and quantification of the wounded areas after wounding the SCC-9 and Cal 27 cells. Scale bar = 100 µm. **(G, H)** Analysis of the invasion and quantification of the SCC-9 and Cal 27 cells. Scale bar = 100 µm. *p < 0.05, **p < 0.01 vs. vector.

To study the effect of cyclin G2 on the proliferation of OSCC, we first constructed stably transfected SCC-9 and Cal 27 cell lines that overexpressed cyclin G2 and control cells that had GFP overexpression (negative control, NC). It was confirmed that the increased expression of cyclin G2 in the cyclin G2 overexpression group through the western blot and RT-qPCR experiments (**Figure 2A**). The CCK8 assay showed that cell **viability** was significantly inhibited in the SCC-9 and Cal 27 cells after cyclin G2 overexpression (**Figure 2B**). Then, the effect of the overexpression of cyclin G2 on the cell cycle was tested in the SCC-9 and Cal 27 cells using flow cytometry. The results showed that the number of G1 cells increased and the number of S cells decreased with cyclin G2 overexpression (**Figures 2C, D**). Thus, cyclin G2 blocks the cell cycle in OSCC cells at the G1/S phase.

To clarify the effects of cyclin G2 on OSCC migration and invasion, we tested the effect of cyclin G2 overexpression in SCC-9 and Cal 27 cells using the wound healing assay and Matrigel transwell assay. The wound-healing rates of the SCC-9 cells in the NC and cyclin G2 groups were 48.67 ± 3.45% and 21.89 ± 4.57%, respectively. The wound healing rates of Cal 27 in the NC and cyclin G2 groups were 38.52 ± 3.45% and 14.15 ± 4.57%. Thus, the wound healing capacity of the SCC-9 and Cal 27 cells was significantly inhibited in the cyclin G2 overexpression group. In addition, cell migration was significantly reduced after cyclin G2 overexpression (**Figures 2E, F**). In the Matrigel transwell experiment, the number of cells passing through the Matrigel and substrate membrane was counted, and the SCC-9 cell numbers for the NC and cyclin G2 groups were 64.2 ± 7.3 and 38.0 ± 4.5, respectively, and the Cal 27 numbers for NC and cyclin G2 groups were 117.6 ± 7.3 and 58.2 ± 4.5. In summary, cyclin G2 overexpression inhibits the migration and invasion abilities of the SCC-9 and Cal 27 cells (**Figures 2G, H**).

### Cyclin G2 Inhibits the Growth and Metastasis of OSCC in Vivo

**Figure 3:**
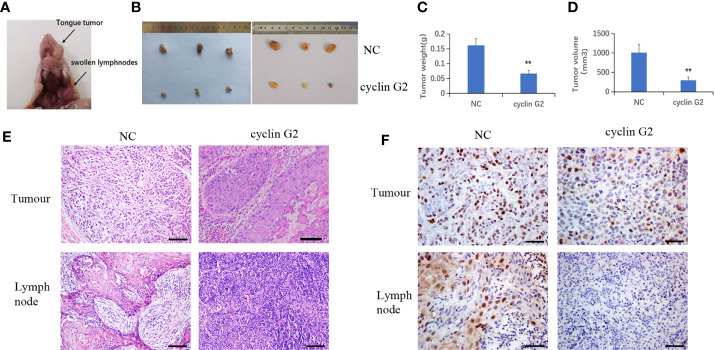
Cyclin G2 inhibits the growth and metastasis of OSCC *in vivo*. **(A)** The tumor in the rear of the tongue and the swollen lymph nodes after 42 days. **(B)** Overall images of the tumors and lymph nodes. **(C, D)** Tumor volumes and weights in the cyclin G2-overexpressing and negative control groups. **(E)** Hematoxylin and eosin staining of the tumor in the rear of the tongue and the swollen lymph nodes. **(F)** Immunohistochemical staining of Ki67 in the oral tumors and lymph nodes. Scale bar = 100 µm. **p < 0.01 vs. vector.

The nude mice were maintained for 42 days after the injection of the SCC9 cells. During that time, tumors formed in the rear of the tongue and the lymph nodes became swollen (**Figure 3A**). The results showed that the volume and weight of the tumors were significantly reduced by the overexpression of cyclin G2 (**Figures 3B–D**). With **Figure 3B** depicting the difference between tumors of NC and overexpress Cyclin G2 while **Figures 3C, D** depict the average differences in weight and volume, respectively. The tumors in the mouth and the swollen lymph nodes were stained with hematoxylin and eosin. OSCC formed in both the cyclin G2 overexpression group and the negative control group. However, the pathological differentiation degree of the tumors was higher in the cyclin G2 overexpression group. The swollen lymph nodes in the cyclin G2 group showed just inflammation, whereas the lymph nodes in the control group showed a significant level of squamous cancer metastasis. We also assessed Ki67 in the two groups of tumors and lymph nodes, and the Ki67 expression was significantly lower in the cyclin G2 group than in the control group (**Figures 3E, F**).

### Cyclin G2 Inhibits the Binding Between IGFBP3 and Integrin by Interacting With IGFBP3

**Figure 4:**
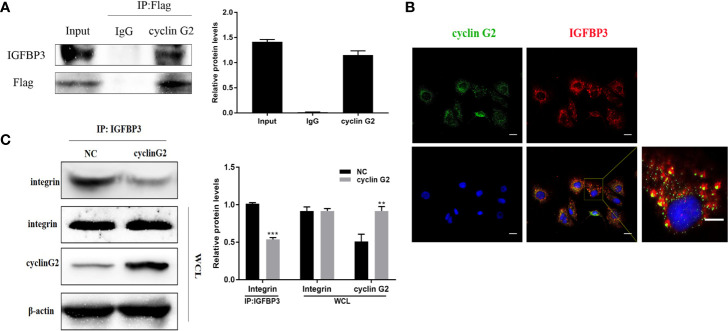
Cyclin G2 may inhibit the binding between IGFBP3 and integrin by interacting with IGFBP3. **(A)** Co-IP analysis of the interaction between cyclin G2 and IGFBP3 in SCC-9 cells. **(B)** Cyclin G2 and IGFBP3 co-localized in the cytoplasm of the SCC-9 cells. **(C)** The combination effects of IGFBP3 and integrin were reduced after cyclin G2 overexpression. **p < 0.01, ***p < 0.001 vs. vector.

To study the molecular mechanism underlying the ability of cyclin G2 to inhibit growth and metastasis in OSCC, we looked for potential protein interactions with cyclin G2 using mass spectroscopy experiments. After screening, we identified IGFBP3 as a possible protein interacting with cyclin G2. We previously published that cyclin G2 also interacted with Dapper 1 and lactate dehydrogenase A, which both further supported the role of cyclin G2 as tumor suppression (18, 19).To validate the findings here, we first extracted proteins from cyclin G2 overexpressing SCC-9 cells, and through the Co-IP experiment, we found that cyclin G2 did indeed interact with IGFBP3 (**Figure 4A**). Next, we performed an immunobifluorescence analysis of cyclin G2 and IGFBP3 in the SCC-9 cells, and cyclin G2 and IGFBP3 were co-located in the cytoplasm (**Figure 4B**). IGFBP3 promotes cell migration and lymph node metastasis in OSCC cells by the requirement of integrin β1 (16). IGFBP-3 binds to the β1-integrin subunits and manipulates the activity of integrin and the integrin receptor complexes and the downstream intracellular signaling pathways (22, 23). However, we found that these combination effects were weakened between IGFBP3 and integrin after cyclin G2 overexpression in the SCC9 cells (**Figure 4C**). This could suggest that the activation effect of IGFBP3 in integrin was inhibited after cyclin G2 overexpression. The integrin receptor complex is inhibited because of the reduced activity of integrin. Additionally, the formation of integrin receptors complexes is recognized to be the critical determinant of signaling from integrin receptors, and thus, the downstream intracellular signaling pathways maybe affected.

### Cyclin G2 Overexpresson Leads to Inhibition of the FAK-SRC-STAT Pathway in Vitro and in Vivo

**Figure 5:**
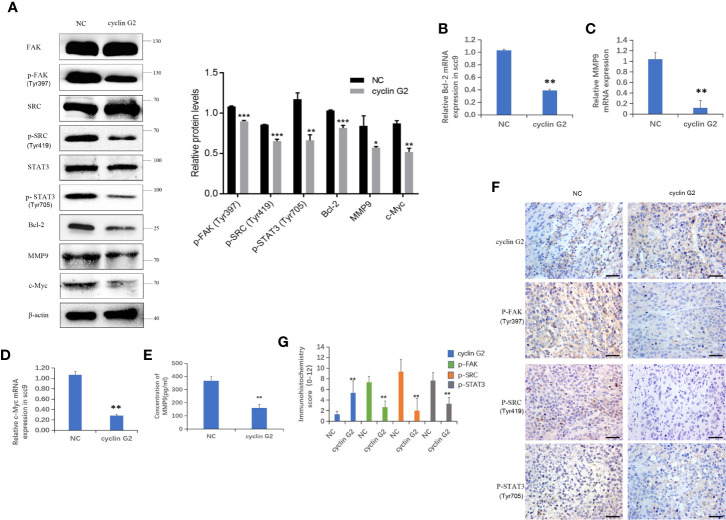
Cyclin G2 inhibits the FAK-SRC-STAT pathway *in vitro* and *in vivo*. **(A)** The protein expressions along with quantification of FAK, P-FAK, SRC, P-SRC, STAT3, P-STAT3, Bcl-2, c-Myc, and MMP9 in the FAK-SRC-STAT pathway. **(B–D)** The relative mRNA expressions of Bcl-2, c-Myc and MMP9. **(E)** Analysis of MMP9 secretion and its quantification in SCC-9 cells. **(F, G)** Immunohistochemical staining and analysis of the expression of cyclin G2, p-FAK, p-SRC and p-STAT3. Scale bar = 100 µm. *p < 0.05, **p < 0.01, ***p < 0.001 vs. vector.

IGFBP-3 modulates the classical integrin-mediated membrane recruitment of FAK and the subsequent activation of SRC (23). We found that the phosphorylation of FAK in the integrin pathway was inhibited after cyclin G2 overexpression in the SCC-9 cells, which could have resulted in the decreased the phosphorylation and activation of the downstream SRC-STAT pathway. STAT3 translocates to the nucleus after its phosphorylation, in order to regulate transcription. We detected the protein expression of the downstream genes *Bcl-2*, *c-Myc* and *MMP9*, and Bcl-2, c-Myc and MMP9 were decreased after cyclin G2 overexpression the levels were quantified and analysed using ANOVA in SPSS (**Figure 5A**). In addition, RT-qPCR revealed that the mRNA levels of *Bcl-2*, *c-Myc*, and *MMP9* decreased after cyclin G2 overexpression (**Figures 5B–D**). Furthermore, we detected MMP9 secretion using an ELISA test. MMP9 secretion was reduced after cyclin G2 overexpression (**Figure 5E**). Finally, the expression of cyclin G2, p-FAK, p-SRC, and p-STAT3 *in vivo* were lower in the cyclin G2 overexpression group (**Figures 5F, G**). Taken together, we concluded that cyclin G2 inhibited the growth and metastasis of OSCC *via* its interaction with IGFBP3.

## Discussion

Cyclin G2 is a tumor suppressor and has a low expression in several cancers (24). In this study, we found that cyclin G2 levels were reduced in clinical OSCC tissue samples and that its expression was negatively correlated with the clinical stage of OSCC. The overexpression of cyclin G2 inhibited the **viability** and metastasis of OSCC cells both *in vitro* and *in vivo*. These findings suggest that cyclin G2 is an inhibitor of OSCC.

The current study demonstrated that the cyclin G2 interacts with IGFBP3. The protein is closely related to the development and metastasis of tumors. In addition, IGFBP3 not only enhances the growth of insulin-like growth factors (IGFS) (25), but also binds to the integrin β1 subunit in an insulin-independent manner. Also, it promotes the formation of a link between the integrin α and β subunits, thereby activating the integrin (26). The integrin receptor regulates the intracellular signaling pathways and the adhesion process of the cells and the extracellular matrix (27). Integrins connect to the actin cytoskeleton and transmit signals from the outside to the inside through focal adhesion multiprotein complexes. Stimulated integrins lead to the activation of the focal adhesion tyrosine kinases, FAK and SRC (28), which, in turn, promotes the activation of numerous downstream signaling pathways that play a pivotal role in cellular processes, such as adhesion, proliferation, migration and survival (29). Nuclear FAK is specifically linked to IGFBP3 through its interaction with Runx1, which leads to cell cycle progression and tumor growth regulation (30). IGFBP3 is overexpressed in a variety of tumors, but its biological significance remains unclear (31–34). The overexpression of IGFBP3 in OSCC promotes cell migration and lymph node metastasis in a manner that depends on integrin beta-1 (16).

Here, we observed that cyclin G2 was able to bind with IGFBP3, and the binding between integrin and IGFBP3 could have been reduced after cyclin G2 overexpression, which impeded the formation of the integrin receptor complex, thereby inhibiting the phosphorylation of FAK and SRC. The Mbd domain of IGFBP3 (mature protein amino acid residues 215–241) interacts with integrin (25). A future study would be aimed to analyze whether cyclin G2 interacts with which part of IGFBP3 domain if it is a part of the Mbd domain of IGFBP3 it will further confirm the competitive role of cyclin G2 and integrin.

FAK is a non-receptor tyrosine kinase and is the central molecule in the transfer process of the integrin-mediated signaling effectuated *via* the Y397 phosphorylation of FAK (35). FAK increases the activity of SRC, which then catalyzes the tyrosine phosphorylation in FAK. Thus, the two proteins are mutually activated (36, 37). The FAK-SRC complex regulates cell growth, differentiation, metastasis, and survival (38). The abnormally activated FAK and SRC proteins are associated with the progression of several types of tumors, including colon cancer, breast cancer, prostate cancer, lung cancer and oral cancer (39–43). Cyclin G2 directly or indirectly inhibits the phosphorylation and activation of FAK and SRC, which affects the activation of downstream STAT3 and inhibits the signal transmission of the FAK-SRC-STAT axis. The regulation of the proliferation, invasion and metastasis of tumor cells allows for the interaction of phosphorylated FAK and SRC with the STAT3 signaling pathway. STAT3 is a transcription factor that is translocated to the nucleus *via* phosphorylation, thereby regulating an array of genes and the expression of proteins associated with growth and metastasis. When the phosphorylation of STAT3 is reduced, its translocation to the nucleus is reduced, which decreases the transcriptional regulation of *Bcl-2*, *c-Myc*, and *MMP9*. While *Bcl-*2 is an anti apoptoicprotein and provides resistance to chemotherapy (44) and *c-Myc* acts as a master regulator of many oncogenic pathways (45) regulating cell growth, differentiation, and malignant transformation, *MMP-9* can cleave many extracellular matrix (ECM) proteins and can regulate ECM remodeling associated with tumor invasion, angiogenesis, and metastasis (46). The mRNA levels of all three genes are greatly reduced in cells overexpressing cyclin G2, thus decreasing the overall oncogenic and metastatic ability of the cells. The inhibition of the STAT3-FAK-SRC axis is implicated in lowering the cancer stem cell load, tumorigenic potential, and metastasis (39). Moreover, this reduction also inhibits MMP-9 and thus tumor invasion (47). Therefore, we assessed the mRNA and protein levels in this pathway and found that the FAK-SRC-STAT axis was inhibited in the presence of cyclin G2 overexpression.

Finally, in nude mice, cyclin G2 overexpression inhibited the growth and metastasis of OSCC tumors. The results from the nude mice showed that cyclin G2 overexpression inhibited the growth and metastasis of OSCC. Interestingly, even though the overexpression of cyclin G2 inhibited the growth and metastasis of OSCC, it was associated with a higher grade compared to the wild type expression. These data suggest that other factors should be considered in conjunction with OSCC overexpression in order to block tumor progression. It would be worth examining human OSCC biopsies of different grades retrospectively and examining cyclin G2 expression in these cases. The advantage of constitutively expressing cyclin G2 is the suppression of tumors. We cannot speculate on the cons as data does not exist as yet. Our lab has raised cyclin G2 knockout mice and has found no changes survival rates or reproduction

This study has several limitations that should be addressed. We showed that cyclin G2, IGFBP3, and integrin show potential protein-protein interactions. However, more details are needed to understand whether these are direct interactions or if other proteins are also involved. In the future, more specific biochemical studies are needed to have a better understanding of these interactions in order to make more solid conclusions about their role in OSCC. In addition, due to the nature of OSCC in our animal model, we had to establish a special feeding method to ensure that we could maintain the mice out to 42 days. In the course of this, 1 mouse did die, and that reduced our sample size from 14 to 12. Whether this model alters the results is unknown. However, for the control mice, we observed the development of OSCC as expected. Thus, this model allowed us to test our hypothesis regarding cyclin G2 overexpression in OSCC to the best of our ability.

**Figure 6:**
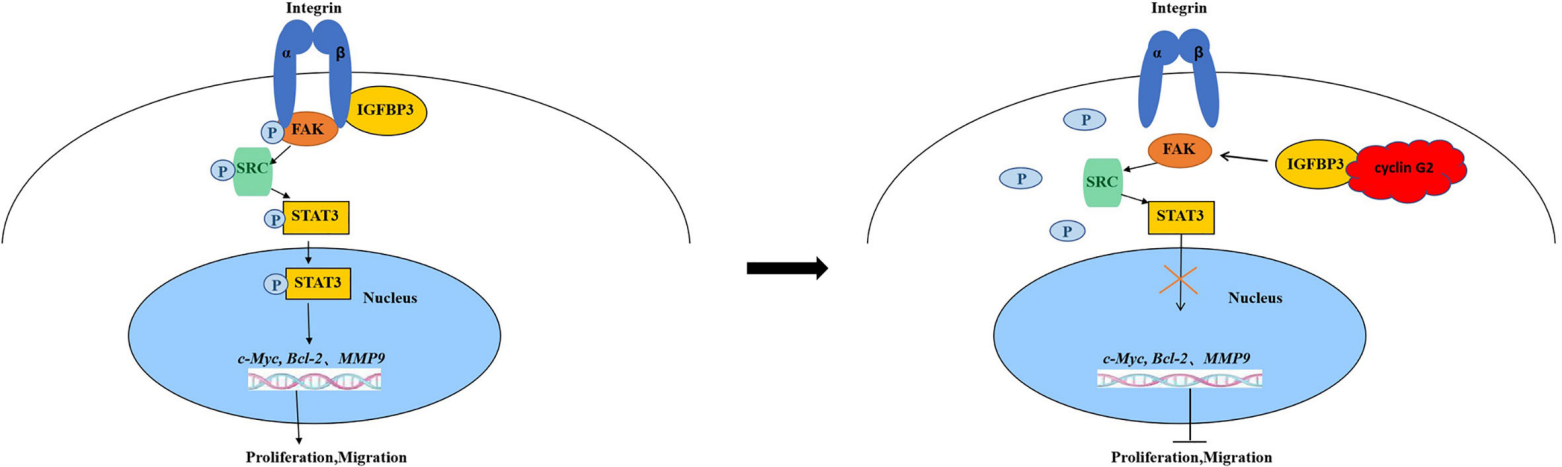
Model for cyclin G2 inhibiting the growth and metastasis of OSCC by binding to IGFBP3 and regulating the FAK-SRC-STAT signal pathway. Cyclin G2 interacts with IGFBP3 and inhibits the binding between integrin and IGFBP3 and the phosphorylation of FAK. FAK dephosphorylation inhibits the FAK-SRC-STAT signal pathway, thereby inhibiting tumorigenesis.

In summary, cyclin G2 binds to IGFBP3 and reduces the phosphorylation level of FAK and SRC and the FAK-SRC-STAT signal transduction. Our findings suggest a new mechanism of cyclin G2 for growth and metastasis in OSCC. We have illustrated the possible mechanisms (**Figure 6**). Therefore, further study of the biological function of cyclin G2 may provide more insight into the molecular basis of the development of OSCC treatment targets.

## Conclusions

Cyclin G2 binds to IGFBP3 and may inhibit the FAK-SRC-STAT signal transduction which could be the primary cause of the growth and metastasis of OSCC. Our findings suggest a new mechanism for the role of cyclin G2 in the development of OSCC. Therefore, cyclin G2 may represent a good target for OSCC treatment.

## Data Availability Statement

The raw data supporting the conclusions of this article will be made available by the authors, without undue reservation.

## Ethics Statement

The animal study was reviewed and approved by The Animal Ethics Committee of China Medical University

## Author Contributions

DW performed all the molecular biology and animal experiments. CZ, SL, XH, and JG assisted in the cell experiments and helped to prepare the experiments; reagents. XZ and QL assisted in the animal experiments. DZ performed the statistical analysis. YL designed the experiments. DW and YL wrote the manuscript. All authors contributed to the article and approved the submitted version.

## Funding

This work was supported by the Foundation of the Education Department of Liaoning Province (LZDK201703, JC2019031), the Development Program of Innovative Research Team, Ministry of Education of China (No. IRT13101) and National Natural Science Foundation of China (No. 82070794).

## Conflict of Interest

The authors declare that the research was conducted in the absence of any commercial or financial relationships that could be construed as a potential conflict of interest.
